# Remote Monitoring Telemedicine (REMOTE) Platform for Patients With Anxiety Symptoms and Alcohol Use Disorder: Protocol for a Case-Control Study

**DOI:** 10.2196/16964

**Published:** 2020-06-24

**Authors:** Núria Pastor, Elizabeth Khalilian, Elsa Caballeria, Danielle Morrison, Unai Sanchez Luque, Silvia Matrai, Antoni Gual, Hugo López-Pelayo

**Affiliations:** 1 HumanITcare - FOLLOWHEALTH SL Barcelona Spain; 2 Grup Recerca Addiccions Clinic, Psychiatry Department Neurosciences Institute, Hospital Clínic Universitat de Barcelona Barcelona Spain; 3 Institut d'Investigacions Biomèdiques August Pi i Sunyer (IDIBAPS), Barcelona, Spain Barcelona Spain; 4 Fundació Clínic per la Recerca Biomèdica Barcelona Spain

**Keywords:** digital health, digital biomarkers, digital phenotype, mental health

## Abstract

**Background:**

Monitoring mental health outcomes has traditionally been based on heuristic decisions, often based on scarce, subjective evidence, making the clinical decisions made by professionals, as well as the monitoring of these diseases, subject to flaws. However, the *digital phenotype*, which refers to the analysis of data collected by measuring human behavior with mobile sensors and smart bracelets, is a promising tool for filling this gap in current clinical practice.

**Objective:**

The objectives of this study are to develop the digital phenotyping of patients with alcohol use disorder and anxiety symptoms using data collected from a mobile device (ie, smartphone) and a wearable sensor (ie, Fitbit) and to analyze usability and patient satisfaction with the data collection service provided by the app.

**Methods:**

We propose to conduct a study among a group of 60 participants split into two subgroups—experimental and control—of 30 participants each. The experimental group will be recruited by physicians from the Hospital Clínic de Barcelona, and the control group will be recruited on a volunteer basis through fliers and social media. All participants will go through pretraining to ensure technological capability and understanding of tasks, then each participant will download the HumanITcare app and will be given a wearable sensor (ie, Fitbit). Throughout the 4-month period, participants will be monitored on a range of factors, including sleep cycle, heart rate, movement patterns, and sociability. All data from the wearable sensors and the mobile devices will be saved and sent to the HumanITcare server. Participants will be asked to complete weekly questionnaires about anxiety, depression, and alcohol use disorder symptoms. Research assistants will ensure timely responses. The data from both sensors will then be compared to the questionnaire responses to determine how accurately the devices can predict the same symptoms.

**Results:**

The recruitment phase was completed in November 2019 and all the data were collected by the end of December 2019. Data are being processed; this process is expected to be completed by October 2020.

**Conclusions:**

This study was created and conducted as a pilot study with the Hospital Clínic de Barcelona, with the purpose of exploring the feasibility of our approach. The study is focused on patients diagnosed with anxiety and alcohol use disorder, but participants were also monitored for depressive symptoms throughout the trial, although these were not part of the initial inclusion criteria. A limitation to our study was the exclusive use of Android smartphones over iOS devices; this could result in a potential selection bias, due to the accessibility and affordability of Android phones as opposed to iOS-based phones. Another limitation might be that reviews of usability and satisfaction could be confounded by factors such as age and familiarity. An additional function that we might add in future studies is the ability for patients to manage their own data.

**International Registered Report Identifier (IRRID):**

DERR1-10.2196/16964

## Introduction

### Background

In the European Union (EU), approximately 165 million people suffer from mental health disorders, namely anxiety, mood disorders, and substance abuse. These mental health disorders have resulted in both direct and indirect global economic costs estimated at US $2.5 trillion in 2010, with the indirect costs (US $1.7 trillion) being significantly higher than the direct costs (US $0.8 trillion), contrasting the trends of other key diseases, such as cardiovascular disease and cancer [1]. Concurrently, problematic alcohol consumption is considered the causal factor of more than 200 diseases, leading to 5.1% of all global diseases and damages being attributed to alcohol abuse. In addition to the health risks this poses, the social and economic damages involved not only for individuals suffering from alcohol abuse but for society in general must be taken into consideration [2].

Monitoring mental health outcomes has traditionally been based on heuristic decisions, often based on scarce, subjective evidence, making the clinical decisions made by professionals, as well as the monitoring of these diseases, subject to flaws [3]. However, the *digital phenotype*, which refers to the analysis of data collected by measuring human behavior with mobile sensors and smart bracelets, is a promising tool for filling this gap in current clinical practice, offering objective evidence in an otherwise subjective field of diagnosis, namely through the utilization of digital biomarkers [4].

Biomarkers are physiological, pathologic, or anatomic characteristics measured objectively and used to evaluate a patient’s health in the status quo by comparing their current biomarkers to ideal, healthy biomarkers. In the wake of advancing technology, *digital biomarkers* are emerging as a new form of tracking patients’ health. Digital biomarkers are considered digital as they utilize sensors and computational tools to collect data. These measurements can be taken outside of a traditional clinical environment using sensors such as mobile devices and wearable sensors [5].

Numerous studies have shown that information collected from behavioral and physiological sensors can be used to improve conventional evaluations of patients with symptoms of anxiety. In addition, patients who use these platforms show higher levels of consistency with their treatment plans than those involved only in conventional practice [6]. There are various examples of the effectiveness of digital phenotype data collection in analyzing anxiety disorders:

1. There exist significant patterns between the time spent in certain places and presence of depression and anxiety, although these relationships were not consistent [7].
2. It has been shown that sensors integrated into smart bracelets measuring conductance and skin temperature can be utilized to reach 78.3% (148/189) accuracy in classifying students into groups of high or low stress levels. Furthermore, the sensors provided a 79% (37/47) accuracy rate for classifying students’ states of mental health [8].
3. Furthermore, analysis from sensors tracking GPS location and incoming and outgoing text messages and calls collected from 54 college students over a 2-week period indicated that levels of social anxiety can be predicted with an accuracy of up to 85% by tracking these variables [9].

Alongside digital tracking of anxiety symptoms, there also exist various apps designed to facilitate the monitoring of alcohol consumption in patients with alcohol use disorder [10-12]. Although more research is needed, these apps have been shown to be effective in increasing patients’ abilities to manage their conditions [10], leading to reduced days of consumption risk [11]. In addition, these tools allow for patients’ usual professional caretakers to monitor their conditions, allowing for more informed care [10]. Therefore, with the REMOTE (Remote Monitoring Telemedicine) Study, we aim to provide valuable insights and knowledge about digital biomarkers in alcohol use disorders and anxiety disorders.

### Study Objectives

Due to emerging research on digital biomarkers and the accuracy of passive monitoring of data in patients with mental health disorders, we propose to conduct the REMOTE Study among a group of 60 participants split into two subgroups of 30 participants each This study will be conducted in the Addictions Unit at the Hospital Clínic de Barcelona. The primary objective of this study is to analyze the digital physiological patterns of two groups of participants—one group with symptoms of anxiety disorder and alcohol use disorder and one healthy control group without these disorders—using data collected from a mobile device (ie, smartphone) and a wearable sensor (ie, Fitbit). The data will be collected from the sensors through the HumanITcare platform. The data will then be compared with the symptoms validated in clinical questionnaires, which participants will complete four times over the course of the study, in order to determine whether the use of sensor data is an efficient and accurate means of presenting objective diagnoses rather than the subjective diagnoses of the status quo.

The secondary objective of this study is to analyze usability and patient satisfaction with the data collection service provided by the app and to explore the feasibility of this approach in clinical practice.

## Methods

### Design

#### Overview

This study is a case-controlled, prospective study consisting of two groups: one group of healthy control individuals who do not have symptoms of alcohol use disorders or anxiety and one experimental group that meets the selection criteria described in the Study Population and Setting section. Participants in both groups will be matched for age and sex, since both can be confounding factors regarding usage patterns of electronic devices. Both groups will receive mobile and wearable sensors that will track their physiological and behavioral activity over the course of 1 month. Both groups will also take the State-Trait Anxiety Inventory (STAI) [13], the Beck Depression Inventory-II (BDI-II) [14], and the Alcohol Use Disorders Identification Test (AUDIT) [15] once per week through the HumanITcare app downloaded on their phones. Participants will be instructed on the use of the app during the initial visit, along with receiving their wearable sensors (ie, Fitbits). The data will be monitored by the research team through an online, encrypted compilation of participants’ data.

The HumanITcare platform allows for the collection of data from the participants; the platform also provides a source of data for the researchers to verify that each participant’s data are being collected appropriately and that the Fitbit devices are working properly. The HumanITcare app that participants download to their phones collects data from the phones and sends them to the HumanITcare platform. As stated above, the app sends notifications to the participants once a week to remind them to complete the questionnaires. Also, it provides a *help* button so they can contact the researchers if needed (see the HumanITcare App section below).

#### Study Population and Setting

The study will be conducted at the Hospital Clínic de Barcelona and is a single-center national study. Experimental participants will be drawn from the outpatient clinic at the Addictions Unit of the Hospital Clínic de Barcelona, and control group participants will be drawn from random volunteers recruited through fliers and social media.

The research team members at the Hospital Clínic de Barcelona Addictions Unit will be tasked with finding eligible subjects for the experimental group. The inclusion criteria for the experimental group participants are as follows: must be between the ages of 18 and 70 years; must have knowledge and daily use of new technologies; must have an alcohol use disorder, based on the Diagnostic and Statistical Manual of Mental Disorders, 5th Edition (DSM-5) [16]; must have significant anxiety symptoms (STAI score >33rd percentile); and must have been informed about the study and signed the data processing consent form. Potential experimental group participants are not eligible if they meet any of the following criteria: their mobile device is not compatible with the Android mobile operating system (OS); they have a diagnosis of affective disorder, based on the DSM-5; they have cognitive deficits that prevent proper participation; or they actively consume other substances, in addition to alcohol, except nicotine.

#### Recruitment

**Figure 1:**
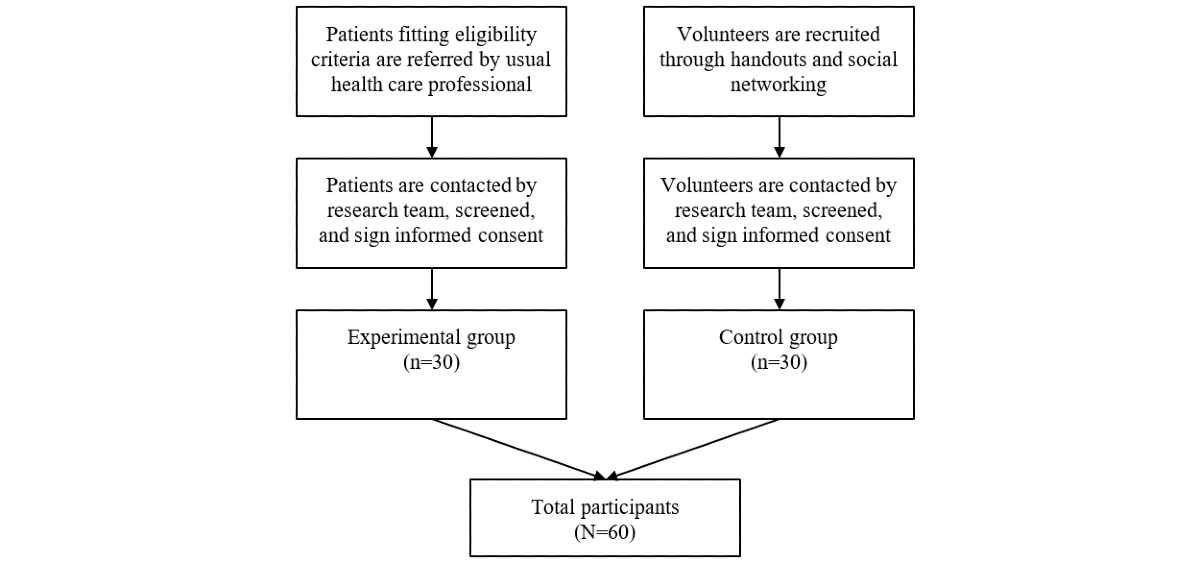
Study recruitment diagram.

There will be a total of 60 participants split between the experimental and control subgroups (see Figure 1).The recruitment of the 30 experimental patients, conducted by psychiatrists and clinical psychologists, will take place in the outpatient clinic and day hospital of the Addictions Unit of the Hospital Clínic de Barcelona. The usual health care professionals for patients with anxiety symptoms will refer the selected patients to the research team who will assess whether the selected patients meet the eligibility criteria. The 30 healthy control participants will be recruited using brochures distributed by the Faculty of Medicine at the University of Barcelona, as well as through social media.

Patients deemed eligible for the study will be contacted by the research team and will have the study explained to them. They will be given a sheet with instructions for proper usage of the Fitbit device (ie, how to load it, how to connect to the app, etc). Informed consent will be given after a full explanation of the trial, including the trial’s use of data assets and potential liabilities.

### Study Procedures

Participants will complete the study phases as described in the following sections.

#### Initial In-Person Visit: First Visit

Once deemed eligible by the research team, participants who meet the requirements for the experimental group, as well as the volunteer control participants, will be called to the Hospital Clínic de Barcelona. The first visit will last approximately one hour and will include an explanation of the project to the participants with time for them to ask questions and clarify any doubts. We will explain to the participants’ their rights and obtain their informed consent in the presence of the research team. We will also explain the measurement of the following variables: age, gender, education level, marital status, symptoms of anxiety (measured with the STAI), symptoms of depression (measured with the BDI-II), evaluation of alcohol use disorder (measured with the AUDIT), use of medication, medical comorbidities, and substance use. Those experiencing current medication or substance abuse will not be considered for the healthy control group.

Once the variables are profiled, each participant will be instructed to download the HumanITcare platform app and will receive their data encryption code and password. The app will use the participants’ mobile phone sensors to collect and consolidate data, which are then sent to the research team for evaluation. A Fitbit device will also be provided and each participant will be informed of their obligation to return the device once the study is completed. For proper operation of the Fitbit, participants will be instructed to access the Fitbit app through their personal email and to specify their age, sex, weight, height, and date of birth. This app complies with data protection laws.

Physiological and behavioral data from the participants’ smartphone devices will be compiled in the HumanITcare servers and will be transmitted, along with the data collected in the Fitbit servers, to a separate server available to the research team, which assigns each participant a unique, randomized character code that functions as a profile name (eg, mt1e45al). The data collected through the HumanITcare app and servers and the Fitbit servers for each participant will be represented by this 8-digit code in order to protect each participant’s privacy.

#### Weekly Assessment of Depression and Anxiety Symptoms

Patients will receive a notification from the HumanITcare app once per week reminding them to respond to the three questionnaires—STAI, BDI-II, and AUDIT [15]—available on the HumanITcare app. This will result in a total of four evaluations throughout the duration of the 1-month study period.

#### Noncontact Visits

Research assistants will perform supervision of participants’ data and will monitor whether participants are answering the three weekly questionnaires through the HumanITcare platform. Research assistants will make reminder calls to participants if they are not responding to the questionnaires, in which case the factors that have influenced nonresponse will be explored. All of this data will be recorded.

#### Final In-Person Visit: Second Visit

The investigator shall summon each participant to the Hospital Clínic de Barcelona to return the borrowed Fitbit device. For participants in both groups, data will be collected regarding user experience with the app through an adaptation of the System Usability Scale (SUS) [17] and the Post-Study System Usability Questionnaire (PSSUQ) [18].

Once the study has been completed, data analysis and data processing will follow. After the statistical analysis is complete, we will proceed to the discussion and interpretation of the results and the writing of a scientific article that outlines the findings.

### HumanITcare App

HumanITcare is a Spanish company that has developed an Internet of Things (IoT) platform (HumanITcare platform) as well as a mobile app (HumanITcare app) for the collection and analysis of the daily data of the participants. The HumanITcare app is an Android and iOS app developed by HumanITcare with the intent of collecting mobile phone sensor data for clinical use. The app continuously collects and stores users’ sensor data within the HumanITcare app servers, automatically uploading the data collected by the sensors to the HumanITcare servers when connected to Wi-Fi. This allows for constant gathering of sensor data regardless of the quality of network service available at the time; this also ensures that mobile data fees are not charged to users.

The HumanITcare platform ensures anonymity for users by utilizing the SHA (Secure Hash Algorithm)-256 hashing algorithm to encrypt the data collected by users’ mobile phone sensors. Furthermore, all of the data collected for each participant are only recognizable by a randomized 8-character code (eg, mt1e45al) assigned to each participant, ensuring that the data are anonymous. The data collected by the HumanITcare app will be evaluated in tandem with data collected by a Fitbit wearable device; however, the app offers a wider breadth of sensors. The passive data types collected by the app and the Fitbit wearable device are represented in Textbox 1.

**Passive sensor data collected by the HumanITcare app and the Fitbit device.**

Mobile phone sensors utilized in the HumanITcare app:

- Accelerometer
- Bluetooth
- Call log
- Text log
- Mobile phone identifier
- GPS
- Power state: battery level
- Power state: charging event
- Power state: screen on or off
- Wi-Fi connection

Fitbit sensors utilized:

- Heart rate monitor
- Sleep monitor
- Accelerometer

### Outcomes

#### Primary Outcome: Sensor Data Accuracy and Efficiency

The symptoms of anxiety and alcohol use disorder presented to the research team will be derived from the algorithm calculated from data collected by the HumanITcare platform (ie, app) and the Fitbit devices. These symptoms will be compared with the diagnoses provided by the status quo, *gold standard* questionnaires—the STAI [13] and the DSM-5 [16]—to determine the feasibility and efficiency of using objective sensor data in place of the current subjective methods of diagnosing mental health disorders. This will be done in a two-step process: first, by determining the relationship between the individual types of data tracked by sensors (eg, distance traveled and sleep schedule) and levels of anxiety and alcohol use disorder symptoms as presented by the questionnaires; and second, by using a mathematical model to estimate questionnaire scores by solely using the data collected by sensors (see Table 1). The primary outcome will be the efficiency of the developed algorithm’s ability to predict disorder symptoms.

**Table 1:** Data collection by sensors compared with questionnaires.

Type of data being collected		Method of data collection
**Sensor data: to be used to calculate estimation algorithm**		
	Sleep pattern	Monitored using a cardiac activity sensor and the Fitbit watch given to participants at the initial visit
	Rapid eye movement (REM) sleep time	Monitored using the Fitbit motion sensor and cardiac activity sensor
	Heart rate	Monitored using the Fitbit sensor
	Step count	Monitored by compiling each participant’s daily step count using an accelerometer, smartphone GPS services, and Fitbit motion sensor
	Distance travelled	Monitored using the GPS services of each participant’s smartphone via the HumanITcare app
	Mobile device usage	Monitored by determining the time frames in which there was a presence or absence of signals from each participant’s device via the HumanITcare app
	Sociability: number of incoming and outgoing calls and text messages	Monitored by the number of incoming and outgoing calls and text messages from each participant’s smartphone via the HumanITcare app
**Questionnaire data: to be tested against estimation algorithm**		
	Self-reported anxiety symptoms	Assessed via the State-Trait Anxiety Inventory (STAI); participants receive STAI scores ranging from 0 (lowest number of symptoms) to 60 (greatest number of symptoms), which are then transformed into percentiles according to age and sex
	Self-reported depression symptoms	Assessed via the Beck Depression Inventory-II (BDI-II); participants receive BDI-II scores ranging from 1 to 63: a score of 0-13 indicates minimal depression, 14-19 indicates mild depression, 20-28 indicates moderate depression, and 29-63 indicates severe depression
	Self-reported alcohol use symptoms	Assessed via the Alcohol Use Disorders Identification Test (AUDIT); participants receive AUDIT scores ranging from 0 to 40: a score of ≥8 is associated with harmful or hazardous drinking and a score of ≥13 in women or ≥15 in men is likely to indicate alcohol dependence

#### Secondary Outcome: System Usability

Satisfaction with and usability of the data collection app will be evaluated after the trial period in order to determine the practicality of the user interface and to determine the practical feasibility of widespread usage of similar apps:

1. Satisfaction with the app will be scored using the PSSUQ [18]. Scores range from 0 (least satisfactory) to 100 (most satisfactory). Participants will be asked to complete the PSSUQ after the data collection period is over.
2. Usability of the mobile app will be scored using the SUS [17]. Scores range from 0 to 100, with a score of ≥68 being considered above average. Participants will be asked to take the SUS after the data collection period is over.

### Data Collection, Management, Security, and Ethics

Treatment, communication, and transfer of personal data of all participants will be adjusted to compliance with the EU Regulation 2016/679 of the European Parliament and of the Council of 27, April 2016, on the protection of individuals with regard to the processing of personal data and the free movement of data. The legal basis that justifies the processing of data is the consent hereby given, pursuant to the provisions of Article 9 of EU Regulation 2016/679.

The data that will be collected from each participant are identified only by a randomized 8-digit code, so they will not include any information that could identify participants. Only the research team members with the right of access to the source data (ie, medical history) may relate the data collected in the study with the clinical history of the patient. The identity of participants will not be available to any other person except for a medical emergency or legal requirement. The Ethics Committee for Research, health authorities, and the personnel authorized by the study sponsor will have access to identifiable personal information when necessary to check data and study procedures but will always maintain confidentiality in accordance with current legislation. Moreover, the project will be conducted in accordance with the Declaration of Helsinki (2013).

### Risk Management Protocol

#### Stage 1: Recruitment

Table 2 shows the factors and challenges associated with participant recruitment, for both the experimental and control groups.

#### Stage 2: Initial In-Person Visit

Table 3 shows the factors and challenges associated with activities of the in-person visit.

#### Stage 3: Monitoring

Table 4 shows the factors and challenges associated with activities during the study duration and symptom monitoring.

#### Stage 4: Second In-Person Visit

Table 5 shows the factors and challenges associated with activities of the second in-person visit.

#### Stage 5: Analysis and Results

Table 6 shows the factors and challenges associated with study analysis and results.

**Table 2:** Difficulties associated with participant recruitment and their correction measures.

Study activity and associated difficulties or risks		Measures to correct or mitigate
**Patient recruitment**		
	Low number of patients recruited or low involvement by health care professionals	Send weekly reminders
	Adult Mental Health Care Center professionals have little familiarity with the questionnaire	Attach the questionnaire in reminder emails for health care professionals
	Delay in the schedule due to slow recruitment	Expand to the maximum period of recruitment
**Control participant recruitment**		
	Low access to paired patient controls	Create database of potential candidates who wanted to participate as controls
	Dependence on the pace of patient recruitment	Provide informative brochures

**Table 3:** Difficulties associated with activities of the in-person visit and their correction measures.

Study activity and associated difficulties or risks		Measures to correct or mitigate
**Presentation protocol and signing informed consent**		
	Participants decide not to participate	Summarize by phone so that they can assess whether to participate (avoid visit if uninterested)
	Participants do not understand the function	Explain in a simple and clear way the protocol and benefits (eg, “measure your sleep...”)
**Patient questionnaires**		
	Patients do not meet the inclusion criteria	Clearly specify the criteria and instruments when explaining the protocol and review medical history
**Download of the app**		
	Lack of storage space on phone	Forewarn the participant before the visit that an app will need to be downloaded
**Instructions of use**		
	Misuse of the app or device	Deliver a clear, visual instruction notebook in the first session Rehearse app use during the visit with patient Provide contact information to call in case of difficulties
**Explaining the follow-up**		
	Participants forget to respond to questionnaires	Prepare a specific calendar for the patients, showing schedule of days to respond to the questionnaires, and schedule a date for the second in-person visit
	Participants forget to wear the device daily	Send weekly reminders

**Table 4:** Difficulties associated with monitoring during the study and related correction measures.

Study activity and associated difficulties or risks		Measures to correct or mitigate
**Correct use of the device**		
	No use	Send notifications encouraging use of the device Call to remind participants and stress the use of the device
	Problems with the server in recording of the data	Perform maintenance of the platform Call to record the daily use if more than 7 days without use
	Defective Fitbit	Provide contact number for technical problems Test devices in advance
	Forget username and/or password	Provide contact number in the instruction book for technical concerns Create database of patients and log-in data Configure the app so that it does not log out the participants
	Problems uploading the Fitbit data	Explain that Bluetooth needs to be enabled for uploading of data; participants should synchronize data every day
**Response to the weekly questionnaires**		
	No response	Send reminders in the form of notifications Review responses from the platform Consider a percentage of 30% as possible dropouts in recruitment
**Loss of the patient from the study**		
	No response to the calls	Consider a percentage of 30% as possible dropouts in recruitment
	Patients reject follow-up	Follow-up call to all patients in the middle of the study to explore difficulties and problems, to encourage, etc

**Table 5:** Difficulties associated with activities of the second in-person visit and their correction measures.

Study activity and associated difficulties or risks			Measures to correct or mitigate
**Visit**			
	Forget to visit	Send reminder call before visit	
**Satisfaction and usability evaluation**			
	Validity of questionnaires	Select valid instruments	
	Bias	Aside from the general questions, include questions according to the number of uses of the bracelet and app	
**Delivery and retrieval of wearables**			
	No return of the device	Stress the necessity of returning the device Communicate that the patient must pay the cost of the device	
	Return in bad condition	Instructions of use Damage will be assessed and, based on impact, the device will be paid for if it is in bad condition	

**Table 6:** Difficulties associated with activities of study analysis and results and their correction measures.

Study activity	Difficulties and risks	Measures to correct and mitigate
Data analysis	Problems with sample variability	Perform preliminary analysis with half of patients Control patients according to degree of use (ie, group according to involvement: a lot, moderate, low, and null)
Dispersion of the results	Scientifically irrelevant data	Assess the usability and satisfaction with the intervention and degree of use Determine association between severity of symptoms and degree of use

### Data Analysis

#### Overview

For each participant, we will collect data through a Fitbit and smartphone device, as well as written data through the form of questionnaires. The devices will offer insight into the physiological responses and changes within participants, which provides a more objective side of the data. For example, the heart rate monitor will report the intensity of one’s heartbeat and could suggest symptoms of anxiety. Additionally, the smartphone GPS will report a participant's movement and, therefore, insight into one’s daily energy or lethargy.

The written responses through questionnaires will give a subjective account of the participants’ anxiety or alcohol use disorder symptoms. An example of this is the STAI, which asks for a scalar rating on one’s feeling of lethargy. Using the data from the Fitbit and smartphone devices, plus the data from the questionnaires, we will determine any correlation.

#### Statistical Methods

The statistical analysis will be carried out as described in this section. Data collected from Fitbit devices, smartphones, and surveys from both groups will be interpreted with descriptive statistics. For the entire group, including all experimental and control participants, the mean and median will be calculated to determine the central tendencies. Next, we will find the standard deviation and range of the data to determine the variability within the dataset. The same process will be done for each group—experimental and control—individually, for the purpose of between-group comparison. The data will also be split and compared based on the source of the data: device information versus survey information.

## Results

The recruitment phase was completed in November 2019 and all the data were collected by the end of December 2019. Data are being processed; this process is expected to be completed by October 2020.

## Discussion

The study is focused on patients diagnosed with anxiety and alcohol use disorder, but participants are also monitored for depressive symptoms throughout the trial, although these were not part of the initial inclusion criteria. Including this data while interpreting results could be a potential confounding factor. However, we would like to emphasize that while we recognize that anxiety disorders and depressive disorders are not clinically synonymous, major studies strongly support the finding that there are high rates of comorbidity between the two. In one study, results showed that 67% of patients with a depressive disorder also had a current anxiety disorder and 75% had a lifetime anxiety disorder [19]. Harvard researchers Jukka-Pekka Onnela and John Torous conducted a similar study using participants diagnosed with major depressive disorder and reported “Patients in this study were not excluded due to comorbidity of additional illnesses,” such as anxiety [6]. Due to this evidence, we believe there is justification for using participants who have symptoms of both. To avoid confusion, future research should prescreen for anxiety as well as depression, and standardize the initial sample. Researchers may also decide to use anxiety and depression interrelated questionnaires, such as the Depression Anxiety Stress Scales (DASS), that take into account the comorbidity of the two disorders, rather than primarily depression-related (ie, the BDI-II) or primarily anxiety-related (ie, the STAI) surveys, in order to improve reliability and validity of patient measures.

A limitation to our study is the exclusive use of Android smartphones over iOS devices. This study was conducted at the beginning stages of our company’s development. At that stage, we did not have the iOS version of the HumanITcare app available. There exists a potential selection bias due to the accessibility and affordability of Android phones as opposed to iOS-based phones. Research shows that Android phones are generally more affordable and financially available to those with a lower socioeconomic status (SES) [20] and that there are higher rates of anxiety and depression present among lower SES populations, possibly due to inferior or limited medical treatment [21]. Thus, this technological criterion may create a biased sample based on SES and the subsequent mental health disorders precipitated by the living conditions of those within this SES. Future research should include a sample of both Android and iOS users to eliminate any confounding variables related to the accessibility of each smart device. Additionally, statistics given by GlobalStats StatCounter show that the mobile OS market-share ratio between Android and iOS is approximately 80:20 [22]. Future research might include a sample that uses Android and iOS devices proportionate to the use in the country to eliminate any confounding variables and to be more accurately representative of the population.

Another limitation might be reviews of usability and satisfaction confounded by factors such as age and familiarity. Prior to the start of the study, researchers ensured that participants understood the tasks and they supplied hard copies of instructions. Researchers from the Hospital Clínic de Barcelona were also present during the training for quality assurance. Nonetheless, it is possible that younger cohorts within the sample are more likely to positively review the usability and satisfaction based on the technological fluency of their generations.

An additional function that we might add in future studies is the ability for patients to manage their own data. We plan to eventually employ an open-database system in which patients can access their own stats and measurements. This may or may not influence the success of the intervention by allowing participants to take notice of their own behavior.

This study was created and conducted as a pilot study with the Hospital Clínic de Barcelona, with the purpose of exploring the feasibility of our approach. The small sample size could be used to calculate the sample size necessary for the most citable outcomes in a follow-up study. Future research will use a larger sample to improve ecological validity. The study experienced one dropout from the experimental group. All original participants remained from the healthy control group.

## Appendix

Multimedia Appendix 1 Evaluation from Comité de Ética de la Investigación con medicamentos del Hospital Clínic de Barcelona.

## Abbreviations

AUDIT: Alcohol Use Disorders Identification Test
BDI-II: Beck Depression Inventory-II
CERCA: Centres de Recerca de Catalunya
DASS: Depression Anxiety Stress Scales
DSM-5: Diagnostic and Statistical Manual of Mental Disorders, 5th Edition
EIT: European Institute of Innovation and Technology
EU: European Union
FEDER: Fondo Europeo de Desarrollo Regional
HS: Headstart
IDIBAPS: Institut d'Investigacions Biomèdiques August Pi i Sunyer
IoT: Internet of Things
OS: operating system
PoC: Proof of Concept
PSSUQ: Post-Study System Usability Questionnaire
REMOTE: Remote Monitoring Telemedicine
RETICS: Red Temática de Investigación Cooperativa en Salud
SES: socioeconomic status
SHA: Secure Hash Algorithm
STAI: State-Trait Anxiety Inventory
SUS: System Usability Scale
