# Supplementary material for: Remote Monitoring Telemedicine (REMOTE) Platform for Patients With Anxiety Symptoms and Alcohol Use Disorder: Protocol for a Case-Control Study
Source: JMIR Res Protoc. 2020 Jun 24;9(6):e16964. doi: 10.2196/16964 (PMC7381016; doi:10.2196/16964)
Supplement: Multimedia Appendix 1 [file resprot_v9i6e16964_app1.pdf]

DICTAMEN DEL COMITÉ DE ÉTICA DE LA INVESTIGACIÓN CON MEDICAMENTOS

NEUS RIBA GARCIA, Secretario del **Comité de Ética de la Investigación con medicamentos del Hospital Clínic de Barcelona**

Certifica:

Que este Comité ha evaluado la propuesta del promotor, para que se realice el estudio:

CÓDIGO:
DOCUMENTOS CON VERSIONES:

Tipo	Subtipo	Versión
Protocolo		Versión 2 de 24/12/2018
Hoja Información de Paciente		VERSIÓN 2 24.12.2018

CIF – G-08431173

TÍTULO: REMOTE "Remote: Monitoring Telemedicine Platform in patients with anxiety symptoms and alcohol use disorder: smartphone and wearable sensor".
PROMOTOR: FOLLOWHEALTH S.L
INVESTIGADOR PRINCIPAL: ANTONI GUAL SOLÉ

y considera que, teniendo en cuenta la respuesta a las aclaraciones solicitadas (si las hubiera), y que:

- Se cumplen los requisitos necesarios de idoneidad del protocolo en relación con los objetivos del estudio y están justificados los riesgos y molestias previsibles.
- La capacidad del investigador y los medios disponibles son apropiados para llevar a cabo el estudio.
- Que se han evaluado la compensaciones económicas previstas (cuando las haya) y su posible interferencia con el respeto a los postulados éticos y se consideran adecuadas.
- Que dicho estudio se ajusta a las normas éticas esenciales y criterios deontológicos que rigen en este centro.
- Que dicho estudio cumple con las obligaciones establecidas por la normativa de investigación y confidencialidad que le son aplicables.
- Que dicho estudio se incluye en una de las líneas de investigación biomédica acreditadas en este centro, cumpliendo los requisitos necesarios, y que es viable en todos sus términos.

Este CEIm acepta que dicho estudio sea realizado, debiendo ser comunicado a dicho Comité Ético todo cambio en el protocolo o acontecimiento adverso grave.

y hace constar que:
1º En la reunión celebrada el día 13/12/2018, acta 22/2018 se decidió emitir el informe correspondiente al estudio de referencia.

Reg. HCB/2018/1121

HOSPITAL CLÍNIC DE BARCELONA
Villarroel, 170 - 08036 Barcelona (España)
Tel. 93 227 54 00 Fax 93 227 54 54
www.hospitalclinic.org

2º El CEIm del Hospital Clínic i Provincial, tanto en su composición como en sus PNTs, cumple con las normas de BPC (CPMP/ICH/135/95)
3º Listado de miembros:

Presidente:
- BEGOÑA GÓMEZ PÉREZ (Farmacéutica Hospitalaria, HCB)

Vicepresidente:
- JOAQUIM FORÉS I VIÑETA (Médico Traumatólogo, HCB)

Secretario:
- NEUS RIBA GARCIA (Médico Farmacólogo Clínico, HCB)

Vocales:
- ITZIAR DE LECUONA (Jurista, Observatorio de Bioética y Derecho, UB)
- MONTSERRAT GONZALEZ CREUS (Trabajadora Social, Servicio de Atención al Usuario, HCB)
- JOSE RIOS GUILLERMO (Estadístico. Plataforma de Estadística Médica. IDIBAPS)
- OCTAVI SANCHEZ LOPEZ (Representante de los pacientes)
- MARIA JESÚS BERTRAN LUENGO (Médico Epidemiólogo, HCB)
- JOAQUÍN SÁEZ PEÑATARO (Médico Farmacólogo Clínico, HCB)
- SERGIO AMARO DELGADO (Médico Neurólogo, HCB)
- JULIO DELGADO GONZÁLEZ (Médico Hematólogo, HCB)
- EDUARD GUASCH I CASANY (Médico Cardiólogo, HCB)
- VIRGINIA HERNANDEZ GEA (Médico Hepatólogo, HCB)
- NURIA SOLER BLANCO (Farmacéutica Hospitalaria, HCB)
- MARINA ROVIRA ILLAMOLA (Farmacéutico Atención Primaria, CAP Eixample)
- JOSE LUIS BLANCO ARÉVALO (Médico Medicina Interna, HCB)
- MIRIAM MÉNDEZ GARCÍA (Abogada, HCB)
- MERCÈ VIDAL FLOR (Enfermera, HCB)

En el caso de que se evalúe algún proyecto del que un miembro sea investigador/colaborador, este se ausentará de la reunión durante la discusión del proyecto.

Para que conste donde proceda, y a petición del promotor,

RIBA GARCIA NEUS - 46540984R

Firmado digitalmente por RIBA GARCIA NEUS - 46540984R
Fecha: 2019.01.25 12:00:25 +01'00'

Barcelona, a 25 de enero de 2019

Reg. HCB/2018/1121

Mod_04 (V4 de 18/06/2018) PR

HOSPITAL CLÍNIC DE BARCELONA
Villarroel, 170 - 08036 Barcelona (España)
Tel. 93 227 54 00 Fax 93 227 54 54
www.hospitalclinic.org

CIF – G-08431173
Generalitat de Catalunya
Departament de Salut
UNIVERSITAT DE BARCELONA
